# Continuous and reversible tuning of the disorder-driven superconductor–insulator transition in bilayer graphene

**DOI:** 10.1038/srep13466

**Published:** 2015-08-27

**Authors:** Gil-Ho Lee, Dongchan Jeong, Kee-Su Park, Yigal Meir, Min-Chul Cha, Hu-Jong Lee

**Affiliations:** 1 Department of Physics, Pohang University of Science and Technology, Pohang 790-784, Republic of Korea; 2 Department of Physics, Sungkyunkwan University, Suwon 440-746, Republic of Korea; 3 Department of Physics, Ben-Gurion University of the Negev, Beer Sheva 84105, Israel; 4 Department of Applied Physics, Hanyang University, Ansan 426-791, Republic of Korea

## Abstract

The influence of static disorder on a quantum phase transition (QPT) is a fundamental issue in condensed matter physics. As a prototypical example of a disorder-tuned QPT, the superconductor–insulator transition (SIT) has been investigated intensively over the past three decades, but as yet without a general consensus on its nature. A key element is good control of disorder. Here, we present an experimental study of the SIT based on precise *in-situ* tuning of disorder in dual-gated bilayer graphene proximity-coupled to two superconducting electrodes through electrical and reversible control of the band gap and the charge carrier density. In the presence of a static disorder potential, Andreev-paired carriers formed close to the Fermi level in bilayer graphene constitute a randomly distributed network of proximity-induced superconducting puddles. The landscape of the network was easily tuned by electrical gating to induce percolative clusters at the onset of superconductivity. This is evidenced by scaling behavior consistent with the classical percolation in transport measurements. At lower temperatures, the solely electrical tuning of the disorder-induced landscape enables us to observe, for the first time, a crossover from classical to quantum percolation in a single device, which elucidates how thermal dephasing engages in separating the two regimes.

The superconductor–insulator transition^1^,^2^ (SIT) in disordered two-dimensional (2D) superconductors exhibits a zero-temperature separatrix between the superconducting and insulating phases^3^,^4^. The associated scaling behavior^5^,^6^ reveals the intrinsic nature of the quantum criticality. Cooper pairs exist even in the insulating phase, which is evidenced by direct observations of the superconducting gap^7^,^8^, as well as the earlier observation of a giant magnetoresistance^4^,^9^, magneto-oscillations^10^, and superfluid correlations^11^. These observations strongly suggest that the loss of pair coherence due to disorder drives the SIT. Several underlying mechanisms for the SIT have been suggested^1^, including the dirty boson picture based on Anderson localization of Cooper pairs^12^, classical^13^ and quantum percolation^14^ of superconducting clusters.

In the dirty boson model^5^,^12^, which assumes that fluctuations of the pair amplitude are negligible, phase fluctuation of the superconducting order parameter destroys global superconductivity. However, some recent reports have pointed out that strong disorder induces amplitude fluctuations to form superconducting islands in the insulating phases, even in homogenously disordered thin films^7^,^15^,^16^,^17^,^18^ (which is also relevant to the Higgs amplitude mode^19^,^20^). These amplitude fluctuations may drive the universality class of the SIT from the disordered boson class to a percolation universality class, governed by the loss of global connection of disordered superconducting islands.

This issue, concerning the interplay between disorder and superconductivity, is underscored by recent experiments, which have reported classical or quantum percolation critical behavior at the SIT in systems with varying degrees of disorder^21^,^22^. The relevance of these two different transitions, classical and quantum percolation, which are governed by different critical exponents, is determined by the question^14^ whether percolating clusters are formed between superconducting islands via either superconducting paths carrying phase-disrupting currents (classical percolation) or via coherent quantum-tunneling links (quantum percolation). Thus, precise control of disorder is crucial to elucidate the interplay between disorder and thermal dephasing, which is responsible for the classical-to-quantum crossover behaviors, and in particular, to differentiate the disorder-induced geometrical effects on the SIT from generic density modulations.

Varying the thickness^1^,^3^ of or annealing^23^ superconducting thin films has been adopted in previous experiments to change the level of disorder. However, this can result in variations in the carrier density as well as the disorder landscape in a non-controllable way. Electrostatic gating has also been employed for 2D superconducting systems as a means of controlling the carrier density while preserving the spatial disorder on an atomic scale in superconducting films^24^, heterostructures of complex oxides^25^, high-*T*_c_ superconductor^26^, and graphene^27^. Here, we utilize electrostatic gating for accurate and reversible tuning of the disorder-induced landscape at energies close to the Fermi level by modulating both the carrier density and the band gap independently, rather than simple carrier density modulation with an uncontrolled fixed disorder.

Compared with deeply buried 2D electronic systems of semiconducting heterostructures or oxide interfaces, graphene is more chemically inert and easily accessible using contact probes. However, the carriers are not strongly localized in monolayer graphene (MLG), even at the charge-neutral point (CNP), where the nominal carrier density vanishes. This is accounted for by the presence of electron–hole puddles^28^,^29^ produced by the disorder potential arising from charged defects on the substrate and/or chemical residues introduced during device fabrication. Since MLG has zero band gap, sufficiently doped bipolar conducting puddles may touch each other [Fig. 1(a)], making the boundaries transmissible by carriers via Klein tunneling. In contrast, in bilayer graphene (BLG), a band gap *E*_g_ opens when an electric field is applied perpendicular to the graphene, separating the charged puddles [Fig. 1(b)], and the transport behavior becomes percolative. This feature of BLG allows a high degree of independent control of both the band gap and the carrier density in a wide range^30^, as shown in Fig. 1(c), and provides flexibility in designing novel devices with controlled conductive behavior by fine-tuning the distance between puddles. Normal percolative transport has been reported in 2D electron gas systems in the low-carrier-density regime^31^, and in MLG nanoribbons with a finite band gap close to the CNP^32^. Similar behavior was observed in this study for the gapped BLG with normal electrodes (see Materials and Methods). As the Andreev-paired carriers were induced by the proximity effect in our dual-gated BLG device, the system precisely simulates a percolative SIT via the puddles of the pairs, the geometry of which is determined by disorder tuning at the Fermi level.

## Results

### Gate-control of superconducting and insulating states

**Figure 1:**
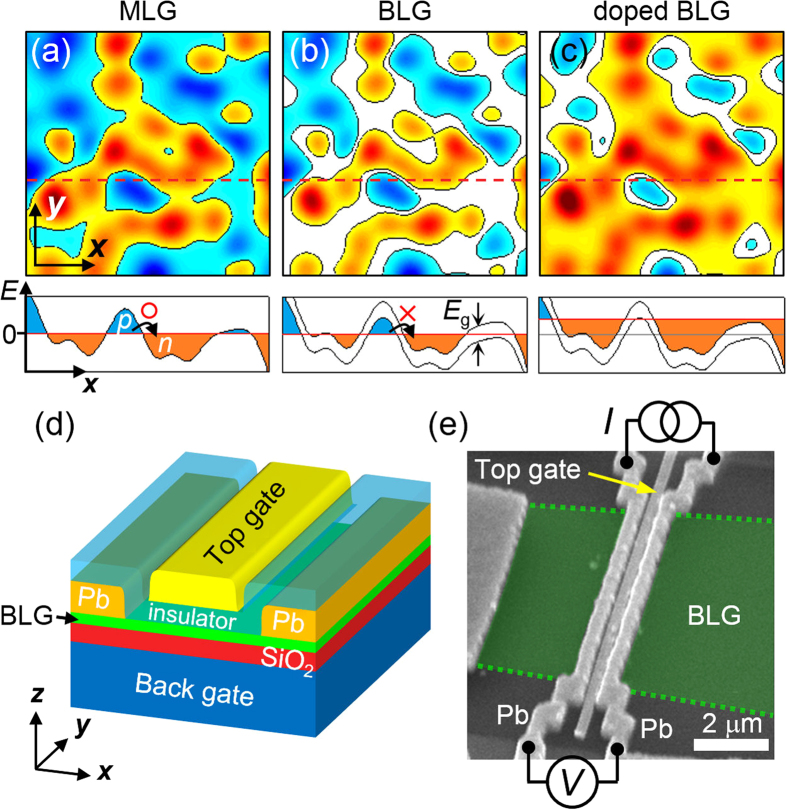
Dual-gated bilayer graphene Josephson junction. (Upper panels) Spatially distributed charged puddles (**a**) in monolayer graphene (MLG) or zero-band-gap bilayer graphene (BLG), (**b**) BLG with a finite band gap *E*_g_ in the charge-neutral state, and (**c**) *n*-doped finite-band-gap BLG. The red, blue, and white represent *n*- and *p*-doped (conducting) and finite-band-gap (insulating) states, respectively. (Lower panels) Cross-sections along the broken red curves in the upper panels, showing the variation in the conduction and valence bands. The solid red curve shows the chemical potential. (**d**) Schematic diagram showing the configuration of the dual-gated BLG Josephson junction. (**e**) Scanning electron microscopy image of the device, illustrating the measurement configuration. The green dotted lines show the location of the BLG.

Figure 1(d) shows a schematic diagram of the configuration of the dual-gated BLG device. A pair of Pb superconducting electrodes was closely attached to a mechanically exfoliated BLG layer, which was sandwiched between the top and bottom gates (see Methods). A scanning electron microscopy image of the device is shown in Fig. 1(e), together with the measurement configuration. The distance (*L*) between electrodes is 0.46 μm and the width (*W*) of the BLG is 7.0 μm. The contact resistances between BLG and Pb electrodes were negligibly small compared to the zero-bias junction resistance, *R* (Supplemental Information, section 1 and Fig. S1). The BLG became superconducting as Andreev-paired carriers formed due to the proximity effect of the superconducting electrodes, along with the consequent Josephson coupling between them^33^,^34^,^35^. The voltages of the bottom gate, *V*_b_, and the top gate, *V*_t_, induced displacement fields *D*_b_ = *ε*_b_(*V*_b_ − *V*_b,0_)/*d*_b_ and *D*_t_ = −*ε*_t_ (*V*_t_ − *V*_t,0_)/*d*_t_, along the 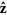 direction, where *ε*’s are the dielectric constants, *d*’s are the thicknesses of the dielectric layers, and *V*_b,0_ (*V*_t,0_) is the charge-neutral voltage offset of the bottom (top) gate due to the initial doping. The difference *D*_density_ = *D*_b_ − *D*_t_ controls the carrier density (or the chemical potential), while the average, *D*_gap_ = (*D*_b_ + *D*_t_)/2, breaks the inversion symmetry of the BLG, opening up a band gap^36^ (Supplemental Information, section 2 and Fig. S3).

**Figure 2:**
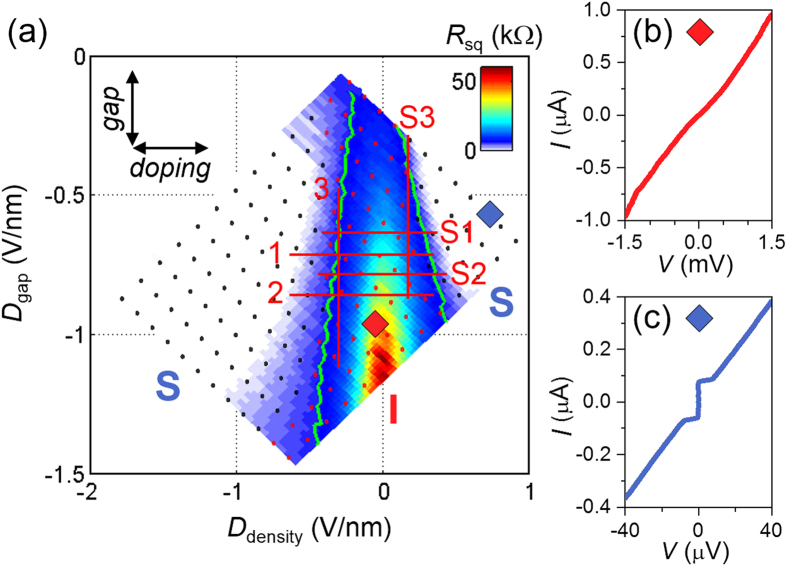
Insulating and superconducting states in an electron-hole puddle system. (**a**) Color-coded square resistance map of the junction as a function of *D*_density_ and *D*_gap_ measured at 50 mK. The diamond symbols indicate representative insulating (red) or superconducting (blue) points of *D*_density_ and *D*_gap_. The green contours correspond to the quantum resistance of Cooper pairs, *R*_Q_ = *h*/4*e*^2^, and separate insulating regions from the superconducting regions. The red curves indicate gate sweep traces 1–3 and S1–S3. Current–voltage characteristics taken at the corresponding points denoted in (**a**) exhibit (**b**) a nonlinear insulating behavior (the red curve) and (**c**) zero-resistance superconducting behavior (the blue curve).

Figure 2(a) shows the square resistance of the junction, *R*_sq_ = *R* × (*W*/*L*), as a function of *D*_density_ and *D*_gap_ measured at a base temperature of *T* = 50 mK. The superconducting and insulating states, marked by black and red symbols, respectively, were determined from the current–voltage (*I*–*V*) characteristics at each set of *D*_density_ and *D*_gap_. The two phases are separated coincidently by the quantum resistance of Cooper pairs, *R*_Q_ = *h*/4*e*^2^ (green contour line) as observed in other systems. On the weakly insulating side, the system exhibited nonlinear insulating *I*–*V* characteristics, as shown in Fig. 2(b), the zero-bias conductance of which is consistent with 2D Mott variable range hopping conduction, *G*(*T*) ~ exp[−(T^*^/*T*)^1/3^], where T^*^ is a characteristic temperature (see Methods). On the superconducting side, *R*_sq_ eventually vanished, and a dissipationless supercurrent branch emerged, as shown in Fig. 2(c), which resulted from the proximity Josephson coupling (Supplemental Information, section 3 and Fig. S4).

### Finite-size scaling analysis on the temperature-dependent behavior

**Figure 3:**
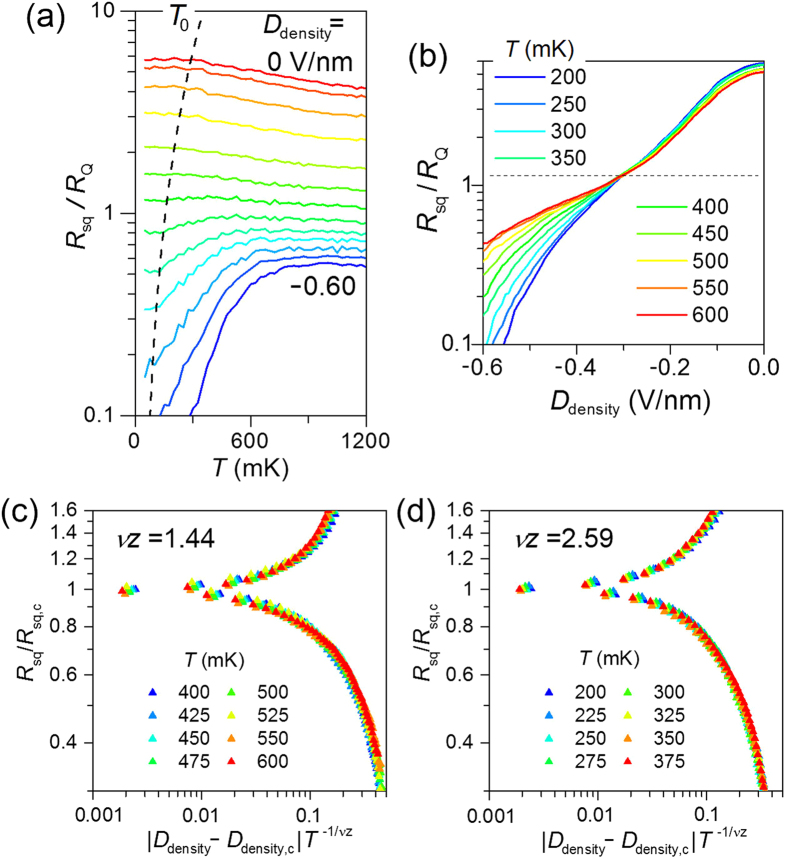
Temperature dependence of square resistance and its scaling behavior. (**a**) Temperature dependence of the square resistance *R*_sq_ with various *D*_density_ for a fixed *D*_gap_ = −0.86 Vnm^*−*1^ plotted with a log-linear axis. *D*_density_ was varied in steps of 0.05 Vnm^*−*1^ from 0 Vnm^*−*1^ (top) to −0.60 Vnm^*−*1^ (bottom). The broken curve indicates the heating-induced crossover temperature *T*_0_, below which the electron temperature and *R*_sq_ saturated. (**b**) The data set in (**a**) plotted as a function of *D*_density_ at various *T*. The horizontal broken line indicates the point of convergence. Finite-size scaling analysis of the *D*_density_-driven superconductor–insulator transition for sweep 2 in Fig. 2(a). For 400 < *T* < 600 mK (**c**), *vz* = 1.44 gave the best data collapse; however, for temperatures of 200 < *T* < 375 mK (**d**), *vz* = 2.59 resulted in the best data collapse.

The temperature dependence of *R*_sq_ at different *D*_density_ ranging from insulating to superconducting phases is shown in Fig. 3(a). It shows no signs of the re-entrance or kink of the resistance at temperatures down to 50 mK, which was commonly observed in granular films. Below the crossover temperature *T*_0_ denoted by the broken line, *R*_sq_ saturated, presumably due to Joule heating of charge carriers. The shift of *T*_0_ to lower temperatures when the heating was reduced (i.e., when *R*_sq_ was smaller) is consistent with the Joule-heating interpretation. In Fig. 3(b), the curves of *R*_sq_ vs *D*_density_ at different temperatures converge on a single point (i.e., *D*_density,c_ ~ −0.3 Vnm^*−*1^) with a corresponding critical square resistance of *R*_sq,c_ ~ 1.1*R*_Q_, which is close to the universal value predicted by the dirty boson model for a low dissipative system.

The SIT behavior is interpreted as a quantum phase transition (QPT), as confirmed by *R*_sq_ vs *D*_density_ data converging to a single finite-size scaling curve^5^,^6^ of the form *R*_sq_ = *R*_sq,c_ *f*(*xT*^−1/*vz*^) close to the critical point [Fig. 3(c,d)]. Here, *f* is a scaling function and *x*  |*D*_density_ − *D*_density,c_| or *x* |*D*_gap_ − *D*_gap,c_| is a tuning parameter. The correlation length exponent ν and the dynamical critical exponent *z* characterize the universality class of the QPT. The data for 400 < *T* < 600 mK exhibit the best collapse, with a critical-exponent product of *vz* = 1.44, which is close to the value *v*_cl_ = 4/3 for classical percolation in 2D^37^ [an exponent of *z* = 1 has been assumed for a system with charged particles^1^, which also appears to be valid in our study, as discussed separately in the bias-field-tuned critical point]. However, at lower temperatures (i.e., 200 < *T* < 375 mK), the best collapse was found with *vz* = 2.59, which is consistent with a quantum percolation transition in 2D with the value *v*_q_ = 7/3 (semi-classically one expects^38^ *v*_q_ = *v*_cl_ + 1). The best estimates of *v*_q_ in the literature^39^ lie in the range 2.3–2.5. We will see below that these values were consistently found in several sweeps with different carrier densities and band gaps. Interestingly, there was a classical-to-quantum crossover at *T*_1_ ~ 400 mK, which will be discussed later in a more quantitative manner. Theoretical studies have predicted^14^,^22^ such a crossover from quantum to classical percolation due to decoherence at a finite temperature. Observations of similar crossover behavior have been reported^22^ for quantum Hall insulator transitions. However, no estimation was provided for the associated change in the electron temperature, *T*_el_, introduced by the bias-induced Joule heating.

### Estimation of T_el_ and the classical-to-quantum crossover

**Figure 4:**
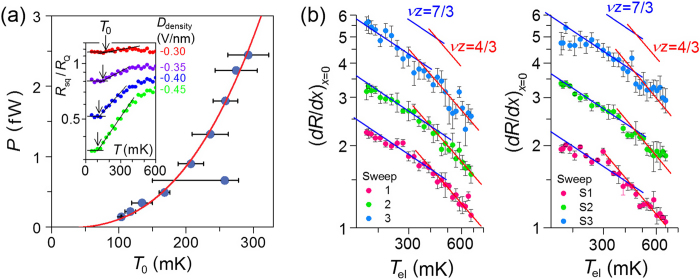
Estimation of electron temperature and temperature dependence of critical exponent products. (**a**) Relationship between the heating-induced crossover temperature *T*_0_ and the dissipative Joule-heating power *P*. The solid curve is a fit to *R*_sq_ data close to the transition in a logarithmic scale. The horizontal lines are guides for the levelling-off of *R*_sq_ and the arrows indicate *T*_0_. (**b**) Electron temperature (*T*_el_)-dependence of resistance slope (*dR*/*dx*)_*x*=0_. For clarity, each data set was shifted vertically by an arbitrary offset. The red and blue linear curves indicate the slope expected with classical and quantum percolation, respectively.

Since Joule heating may seriously affect the behavior of the QPT, in particular, close to the lowest measurement temperature, we carried out an in-depth quantitative analysis of *T*_el_. *T*_el_ saturated to a temperature *T*_0_ as the phonon temperature *T*_ph_ (i.e., the measurement temperature) approached the base temperature, i.e., *T*_el_ = *T*_0_ when *T*_ph_ = 50 mK. The dissipative power *P* = *I*^2^*R* at *T*_ph_ = 50 mK was estimated from the saturated resistance *R* and the root-mean-square (r.m.s.) bias current of *I* = 1 nA, which exhibited a power-law dependence on *T*_0_, as shown in Fig. 4(a), along with the fit to 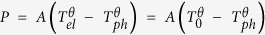 with *T*_ph_ = 50 mK. The fitting parameters were the electron–phonon coupling exponent *θ* = 2.8 ± 0.1 and the coefficient *A* = 77 ± 14 fW∙K^−2.8^, where *T*_0_ was estimated to be *T*_0_ = 160 mK at the SIT point of *R*_sq_ ~ 1.1*R*_Q_ (Supplemental Information, section 4 and Fig. S5). The value of *θ* was consistent with the recently observed value in MLG^40^ in millikelvin regime.

With the electron temperature described by 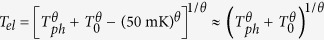, we now discuss the temperature dependence of the critical exponents in detail. The exponent product *vz* can be evaluated from the slope of a double logarithmic plot of 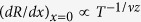 vs *T*, as shown in Fig. 4(b), for each gate sweep of the *D*_density_-tuned (sweep 1, 2, S1, and S2) and *D*_gap_-tuned (sweep 3 and S3) SIT. Note that, in this plot, the heating effect is excluded by replacing the measurement temperature by the electron temperature with *T*_0_ = 160 mK. For all gate sweeps, for *T* > 400 mK, the slope is described well by classical percolation (red line), whereas for *T* < 400 mK, the slope is consistent with the quantum percolation model (blue line). Successful elimination of the Joule heating effect in this study made it possible to identify a crossover between classical and quantum percolation, with the temperature as a tuning parameter for the decoherence.

### Finite-size scaling analysis for bias electric field

**Figure 5:**
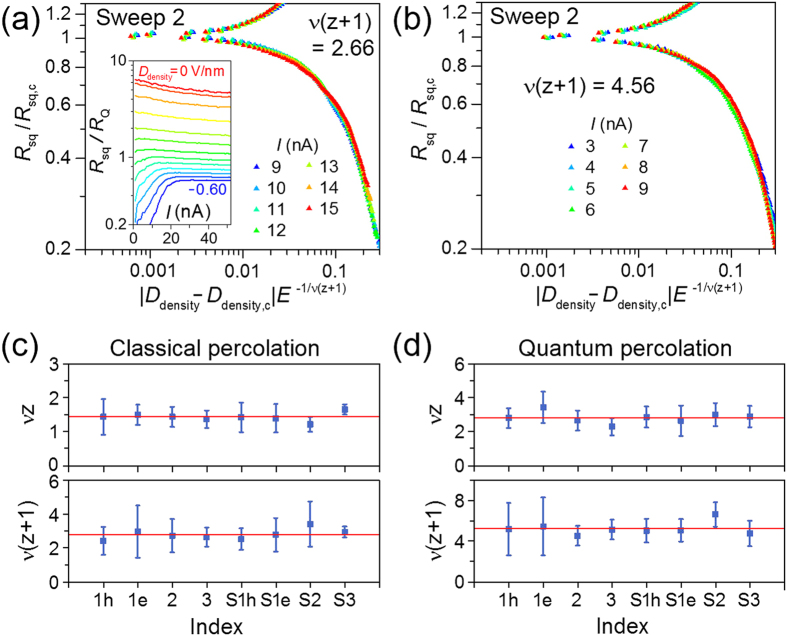
Scaling of square resistance for different electric fields and critical-exponent products for various gate sweeps. Finite-size scaling analysis of electric field dependences of *D*_density_-driven transition for the gate sweep 2, which yields the best collapse with *v*(*z* + 1) = 2.66 for *I* = 9–15 nA in (**a**) or *v*(*z* + 1) = 4.56 for *I* = 3–9 nA in (**b**). Inset of (**a**), bias current dependence of *R*_sq_ at different doping levels (*D*_density_, from 0 to −0.60 Vnm^*−*1^, in steps of 0.05 Vnm^*−*1^). Critical-exponent products *vz* and *v*(*z* + 1) evaluated at various critical points for (**c**) the classical percolation regime and (**d**) the quantum percolation regime. Red lines represent average values. The characters ‘h’ and ‘e’ in the sweep indices stand for the hole and electron side, respectively.

Similar to the temperature dependence, the bias current (*I*) dependence of *R*_sq_ is also differentiated into two phases as shown in the inset of Fig. 5(a), such that *R*_sq_ decreases (increases) with lowering *I* in the superconducting (insulating) phase. Here, we emphasize that the value of *θ* satisfies the ‘safety’ criterion^6^ 2/*θ* > *z*/(*z* + 1), where *z* = 1, for the intrinsic fluctuations being dominant in the Joule-heating effect. This allowed fitting of the critical-exponent product *v*(*z* + 1) for both the classical and quantum percolation regions from the scaling behavior as a function of the bias electric field. The finite-size scaling analysis with the electric field (*E*) in Fig. 5(a,b) provides additional information of *v*(*z* + 1), because the *E* dependence of *R*_sq_ has the form *R*_sq_ = *R*_sq,c_ *g*[*xE*^−1/*v*(*z*+1)^] near the critical point^6^,^41^. Here, *g* is another scaling function. Similar to the *T*-varying scaling in Fig. 3(c,d), *E*-varying scaling also gives two different values of *v*(*z* + 1) depending on the bias current range. For *I* = 9–15 nA, the best scaling was obtained with *v*(*z* + 1) = 2.66, which is close to the value of classical percolation [*v*(*z* + 1) = 8/3]. But, for the lower bias current range of *I* = 3–9 nA the best fit was obtained with *v*(*z* + 1) = 4.56, which is close to the value of quantum percolation [*v*(*z* + 1) = 14/3].

### Products of critical exponents, *vz* and *v*(*z* + 1)

*v*(*z* + 1), together with *vz* from the *T*-varying scaling, allows an independent determination of the critical exponents^41^ of *v* and *z*. We investigated several critical points for both of *D*_density_–driven and *D*_gap_–driven SIT as indicated in Fig. 2(a). For each gate sweep, we performed scaling analysis for both the temperature and electrical field dependences to evaluate the critical-exponent products of *v*z and *v*(z + 1), respectively. We summarized all the critical-exponent products in Fig. 5(c,d) for both of the classical and quantum percolation regimes. The corresponding scaling results for the classical percolation regime are shown in supporting information (Supplemental Information, section 5 and Figs S7 and S8). At higher temperatures (*T* > 400 mK) or for higher electric fields (*I* > 9 nA), with the averaged values of *vz* = 1.44 ± 0.13 and *v*(*z* + 1) = 2.81 ± 0.31 for all different gate sweeps (sweeps 1–3 and S1–S3), we get *v* = 1.37 ± 0.34 and *z* = 1.05 ± 0.27. This result supports the SIT of charged bosons (Cooper pairs) in the classical percolation universality class, which is consistent with the percolative transport nature of carriers through charged puddles in BLG at *T* = 4.2 K. At lower temperatures (*T* < 400 mK) or lower electric fields (*I* < 9 nA), the averaged values of *vz* = 2.83 ± 0.33 and *v*(*z* + 1) = 5.25 ± 0.63 give *v* = 2.42 ± 0.71 and *z* = 1.17 ± 0.37, which support the quantum percolation universality class for SIT.

## Discussion

It is rather surprising that the BLG layer in the narrow region between the superconducting electrodes show the finite-size scaling behaviour of a 2D SIT, which is usually observed in homogeneous 2D systems. We believe that the temperature range of our transport measurements was sufficiently low as to allow the critical behaviour of the correlation length as a function of temperature. The observed temperature-dependent finite-size scaling was then governed by the temporal scale associated with the system temperature without apparent influence of the spatial scale of our device on the transition characteristics. Apparently, the spatial correlation length remained limited at finite temperatures (i.e., shorter than the spacing between the superconducting electrodes) as to neglect the effects arising from possible inhomogeneity of carrier transport or finite size of our system.

Our BLG devices provide a unique method to investigate the underlying mechanisms of SITs via accurate and reversible control of disorder. Electrical gating changed the average spacing between proximity-induced superconducting puddles to drive the QPT as Andreev-reflected bound pairs at the Fermi level establish long-range coherence via percolative paths to yield the critical power-law behavior of percolation with negligible thermal intervention. At lower temperatures than the range of classical percolation behavior, direct control of the disorder enabled us to estimate the effective electron temperature and consequently to identify the crossover between classical and quantum percolation in a single device. Previously, these two regimes have only been obtained in separate systems belonging to weak and strong disorder regimes^21^. Our proximity-coupled BLG system demonstrates that it is an exceptionally useful platform to study disorder-induced QPTs.

## Methods

### Device fabrication

**Figure 6:**
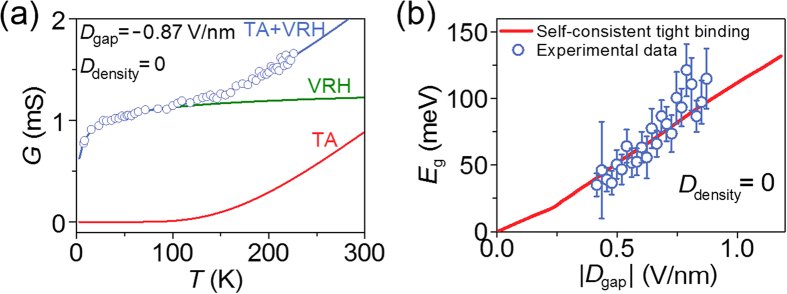
Thermal activation and variable range hopping conduction at the charge neutrality point. (**a**) Temperature dependence of conductance *G* at *D*_gap_ = −0.87 V/nm and *D*_density_ = 0 (charge neutrality point). The data were taken by ac measurements at zero dc bias and are fitted to the parallel conduction model (blue line), which consists of thermally activated conduction (red line) and variable-range-hopping conduction (green line). Best-fit parameters are *E*_g_ = 115 ± 23 meV and *T*_h_ = 1.92 ± 0.59 K. (**b**) Band gap *E*_g_ estimated from the temperature dependence of conductance is plotted as a function of |*D*_gap_|. Red line represents the prediction by self-consistent tight-binding calculation.

Fabrication of the bilayer-graphene Josephson-junction devices relied on mechanical exfoliation of graphene^42^ on a highly doped silicon substrate, which was capped with a 300-nm-thick silicon oxide layer to form a bottom gate dielectric (*d*_b_ = 300 nm, *ε*_b_ = 3.9). Bilayer graphene was identified via optical contrast (Supplemental Information, section 6 and Fig. S9). Superconducting electrodes were defined using electron beam lithography and thermal evaporation of Pb_0.9_In_0.1_ onto the bilayer graphene. Indium was added to minimize the grain size and the surface roughness^35^. The junction area was covered with cross-linked poly(methyl methacrylate) (PMMA)^43^,^44^, which formed a dielectric layer for the top gate (*d*_t_ ≈ 43 nm, *ε*_t_ = 4.5). A Ti/Au top-gate electrode stack (where the layers were 5- and 145-nm-thick, respectively) was deposited and accurately aligned to cover most (~90%) of the junction area (Supplemental Information, Fig. S2). This allowed uniform gate control over the entire junction area. The thickness of the top gate dielectric, *d*_t_, was determined from the shift of the resistance maximum of *V*_t_ by the modulation of *V*_b_. *V*_b,0_ and *V*_t,0_ were determined by comparing the band gap, which was estimated from the temperature dependence in Fig. 6.

### Low-noise measurements

The sample was maintained in thermal contact with the mixing chamber of a dilution fridge (Oxford Kelvinox AST) and cooled to a base temperature of 50 mK. Electrical measurement lines were filtered by a combination of two-stage low-pass RC filters (with a cut-off frequency of ~30 kHz) mounted at the mixing chamber and pi-filters (with a cut-off frequency of ~10 MHz), which were at room temperature. We used a conventional lock-in technique with a bias current amplitude 1 nA r.m.s. at a frequency of 13.33 Hz for the temperature-dependent measurements, and a direct-current bias for the bias-field-dependent measurements.

### Temperature dependence of conductance at CNP

At the charge neutrality point (*D*_density_ = 0), the Fermi level is placed in the middle of the bandgap *E*_g_. Then, the conduction occurs with thermally activated carriers, providing the temperature (*T*) dependence of conductance, *G*_TA_(*T*) = *G*_TA,0_ exp(−*E*_g_/2*k*_B_*T*), with Boltzmann constant *k*_B_. However, in disordered bilayer graphene, bandgap is filled with the localized states such as conducting electron and hole puddles so that the carriers can hop across these states. Hopping transport is more pronounced at lower temperatures where the thermal activation (TA) is exponentially suppressed. As shown in Fig. 6(a), low-temperature conductance agrees with variable range hopping (VRH) model in two dimensions, *G*_VRH_(*T*) = *G*_VRH,0_ exp[−(*T*_h_/*T*)^1/3^], whereas high-temperature data agree with the TA conduction. The measurement was done at temperatures above ~7 K, with the Pb electrodes in the normal state. The charge neutrality point for the top gate was estimated to be *V*_t,0_ = −6.0 V, where the |*D*_gap_| dependence of resultant fitting parameter *E*_g_ agrees with the theoretical prediction of self-consistent tight-binding calculation as shown in Fig. 6(b). Similar TA+VRH transport properties were experimentally investigated in dual-gated bilayer graphene^45^. We could not directly determine *V*_t,0_ as it was beyond the charge-leakage voltage of the top gate. However, the uncertainty in the determination of *V*_t,0_ gives additional offsets to *D*_gap_ only but does not affect the scaling analysis discussed in the text.

### Percolation transport in gapped bilayer grapheme

**Figure 7:**
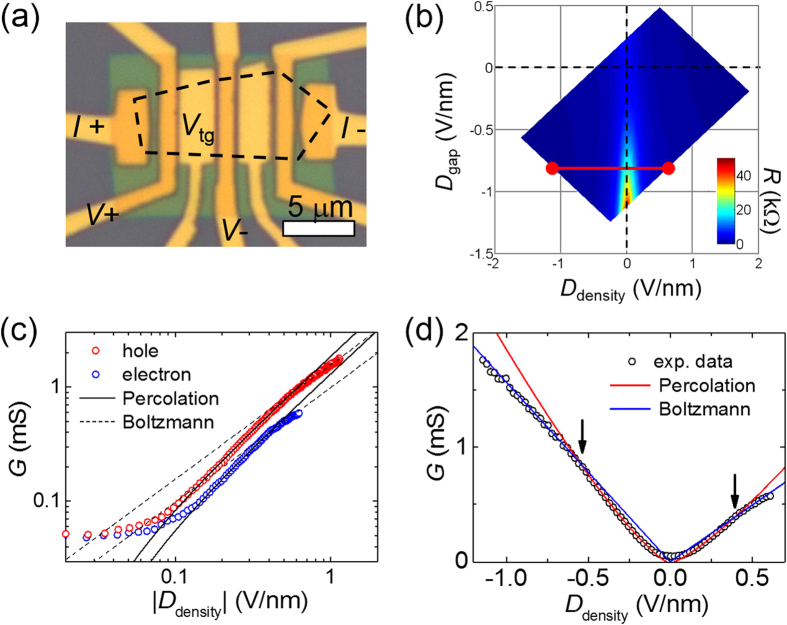
Percolation transport behavior in the gapped bilayer graphene. (**a**) Optical image of the dual-gated bilayer graphene device with measurement configuration. (**b**) Resistance map as a function of *D*_density_ and *D*_gap_ measured at 4.2 K. (**c**) Log-log plot of conductance as a function of |*D*_density_| in the hole side (red symbols) and electron side (blue symbols). Best fits to the percolation behavior (solid lines) in the range of 0.1 V/nm < |*D*_density_| < 0.5 V/nm gives the critical exponent of *δ*^h^ = 1.25 ± 0.02 and the critical carrier density of 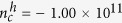 cm^−2^ i*n* the hole side, and *δ*^e^ = 1.25 ± 0.05 and 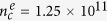 cm^−2^ i*n* the electron side. Dotted lines represent the Boltzmann transport behavior for highly doped state (|*D*_density_| > 0.5 V/nm). (**d**) Linear plot of the same data and the fitting lines of (**c**). Arrows indicate the crossover between Boltzmann and percolation transport regimes.

Carrier density inhomogeneity in two-dimensional (2D) GaAs semiconducting systems induces the percolative metal–insulator transition (MIT) in the low carrier density regime^31^,^46^. Similarly, graphene which has inhomogeneous charge puddles is also expected to exhibit the percolative MIT if a bandgap is introduced to separate the electron band from the hole band. For example, S. Adam *et al.*^32^ fabricated graphene into a nanoribbon structure to open a bandgap in graphene and demonstrated a 2D MIT of the classical percolation universality class. There is also theoretical prediction of percolation behavior for bilayer graphene with a finite bandgap^47^. In our case, a vertical electric field opened a bandgap in bilayer graphene. We investigated transport properties of bilayer graphene in the presence of the superconducting proximity effect and analyzed them in the frame of percolative superconductor–insulator transition. To support the percolative transport characteristics in gapped bilayer graphene in the absence of superconductivity, we fabricated and performed control experiments with a device consisting of dual-gated bilayer graphene in contact with non-superconducting Ti/Au electrodes. Optical image of the device and the measurement configuration are shown in Fig. 7(a). While injecting current (*I* = 1 nA r.m.s.) from *I*^+^ to *I*^−^, voltage drop between *V*^+^ and *V*^−^ was measured as a function of bottom (*V*_b_) and top (*V*_t_) gate voltages at the base temperature of *T* = 4.2 K. According to the definition of *D*_density_ and *D*_gap_, a resistance map is plotted as a function of *D*_density_ and *D*_gap_ in Fig. 7(b). *D*_density_ represents the carrier density (*n* = 5.52 × 10^12^ cm^−2^ × *D*_density_∙V^−1^nm) accumulated by the electrical gates, while *D*_gap_ determines opening of bandgap (*E*_g_) in the bilayer graphene. *D*_density_ dependence of conductance (*G*) at a fixed *D*_gap_ = −0.8 V/nm [along the red line in Fig. 7(b)] is plotted in Fig. 7(c) on log-log scale. The bandgap is estimated to be *E*_g_ ~ 90 meV according to the self-consistent tight-binding model^30^,^48^. There appears three transport regimes depending on the *D*_density_ in both electron and hole sides. In a highly doped state (|*D*_density_| > 0.5 V/nm), Fermi level exceeds the bandgap (|*E*_F_| > 100 meV) so that the system is expected to be in the *Boltzmann transport regime*^49^ where 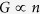. In the range of 0.1 V/nm < |*D*_density_| < 0.5 V/nm, best fits to the *critical behavior* 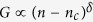 give exponents *δ*^h^ = 1.25 ± 0.02 in the hole side and *δ*^e^ = 1.25 ± 0.05 in the electron side, where *n*_c_ is the critical carrier density. They are close to the theoretical prediction *δ* = 4/3 for 2D classical percolation universality class. Near the charge neutrality point, |*D*_density_| < 0.1 V/nm, *G* deviates from the percolation behavior and does not converge to zero but *becomes saturated*. This is because electron and hole puddles remain conducting even though the average carrier density vanishes at *D*_density_ = 0. Figure 7(d) shows the same data and corresponding fitting lines of Fig. 7(c) on linear scale. The linear relation between *G* and *n* in the Boltzmann transport regime (blue lines) and the crossover between percolation and Boltzmann transport regimes (arrows) are more pronounced.

## Additional Information

**How to cite this article**: Lee, G.-H. *et al.* Continuous and reversible tuning of the disorder-driven superconductor-insulator transition in bilayer graphene. *Sci. Rep.* **5**, 13466; doi: 10.1038/srep13466 (2015).

## Supplementary Material

Supplementary Information
