# Supplementary material for: Continuous and reversible tuning of the disorder-driven superconductor–insulator transition in bilayer graphene
Source: Sci Rep. 2015 Aug 27;5:13466. doi: 10.1038/srep13466 (PMC4550864; doi:10.1038/srep13466)
Supplement: Supplementary Information [file srep13466-s1.pdf]

Supplementary Information

Continuous and reversible tuning of the disorder-driven superconductor–insulator transition in bilayer graphene

Gil-Ho Lee[†1], Dongchan Jeong[‡1], Kee-Su Park[2], Yigal Meir[3], Min-Chul Cha[§4], and Hu-Jong Lee[§1]

[1]Department of Physics, Pohang University of Science and Technology, Pohang 790-784, Republic of Korea

[2]Department of Physics, Sungkyunkwan University, Suwon 440-746, Republic of Korea

[3]Department of Physics, Ben-Gurion University of the Negev, Beer Sheva 84105, Israel

[4]Department of Applied Physics, Hanyang University, Ansan 426-791, Republic of Korea

[†]Present address: Department of Physics, Harvard University, Cambridge, MA 02138, USA

[‡]Present address: Semiconductor R&D Center, Samsung Electronics Co. LTD., Hwasung 445-701, Republic of Korea

[§]Correspondence and requests for materials should be addressed to M.-C.C. (email: mccha@hanyang.ac.kr) or to H.-J.L. (email: hjlee@postech.ac.kr).

1. Contact resistance

Since the measured device resistance includes the contact resistance (R_c), we estimated R_c using a four-probe measurement scheme shown in Fig. S1(a). The measured four-probe contact resistance [$R_{c,4p} = (V_+ - V_-)/I_{bias}$] of the left and right contacts were –3 Ω and –4 Ω, respectively. Here, V_+ and V_- are the electrical potential of the two electrodes and I_{bias} is the bias current between I_+ and I_- contact leads. The negative value of $R_{c,4p}$ in the cross-junction geometry can be understood as that R_c is much smaller than the electrode resistance R_{line} (~ 7 Ω), resulting in non-uniform current flow along the junction #1. When R_c is sufficiently larger than R_{line}, bias current flows uniformly through the junction along the vertical direction and each top and bottom electrode becomes equipotential. This results in voltage difference $\Delta V = V_+ - V_-$ to be positive [Fig. S1(b)] and $R_{c,4p}$ well represents R_c. However, when R_c is sufficiently smaller than R_{line}, the electrode on top and the graphene layer at bottom behave as a single piece with the current flow becoming nonuniform along the junction. In this case, V_- gets higher than V_+, which leads to a negative value of $R_{c,4p}$. This feature is confirmed in the numerical simulation for different values of R_c in Fig. S1(c) and S1(d). Simulation was done by commercial package COMSOL Multiphysics with the same geometrical and electrical parameters of the device. As R_c gets smaller than R_{line}, $R_{c,4p}$ becomes negative and saturated to the value of $-R_{line}$ [Fig. S1(e)], which is close to the experimentally measured value of $R_{c,4p}$. This ensures that R_c of our device was an order of a few ohms, which was negligible compared to the device resistance (a few hundreds ohms).

2. Coordinate transformation of the resistance map

Experimentally, we constructed a resistance map as a function of the bottom gate and top gate voltages (V_b and V_t, respectively) as shown in Fig. S3(a). The carrier doping and the gap opening of the bilayer graphene were exclusively determined by the parameters $D_{density}$ (= $D_b - D_t$) and D_{gap} [= ($D_b + D_t$) / 2], respectively, with $D_b = \varepsilon_b(V_b - V_{b,0})/d_b$ and $D_t = -\varepsilon_t (V_t - V_{t,0})/d_t$. Here, ε is the dielectric constant, d is the thickness of dielectric layers, and $V_{b,0}$ ($V_{t,0}$) is the charge-neutral gate voltage of the bottom (top) gate due to the initial environmental doping. Thus, for convenience, we transformed the coordinate for the resistance map from the (V_b, V_t) basis system to the ($D_{density}$, D_{gap}) basis system as shown in Fig. S3(b).

3. Josephson coupling in the superconducting phase

When the square resistance becomes smaller than the quantum resistance, the superconducting phase emerges in the region of bilayer graphene layer. As discussed in the main text, the superconducting phase is induced by the proximity effect from the superconducting electrodes. In this section, we present the genuine Josephson coupling via the bilayer graphene layer, confirmed by microwave irradiation and applying perpendicular magnetic fields on the bilayer-graphene Josephson junction. When a microwave was irradiated on the Josephson junction, the beating of ac voltage and the ac Josephson effect generated equidistant voltage steps in the current–voltage characteristics [Fig. S4(a)], which is known as Shapiro steps[1]. In Fig. S4(b), the voltage step size ΔV shows highly linear relationship with the irradiated microwave frequency f_{mw} as $\Delta V = hf_{mw}/2e$ with Planck's constant h and electron charge e.

Microwave amplitude ($P^{1/2}$) dependence of differential resistance (dV/dI) with fixed f_{mw} = 5 GHz is plotted in Fig. S4(c). Shapiro steps (dV/dI = 0) shows well-behaving Bessel-function-like oscillation as a function of $P^{1/2}$.

Another unique feature of Josephson junction is periodic oscillation of critical current (I_c) with applied perpendicular magnetic field (B), which is known as Fraunhofer pattern[1]. When the magnetic flux $\Phi = BA_{eff}$ threading the effective junction area A_{eff} becomes an integer multiple of magnetic flux quantum $\Phi_0 = h/2e$, I_c drops to zero except for B=0. Here, $A_{eff}=W(L+2\lambda_L)$ with taking into account of the London penetration depth λ_L of the superconducting Pb-In electrodes. W = 7.0 μm is the width and L = 0.46 μm is the length of the Josephson junction. In Fig. S4(d), the B dependence of I_c clearly manifests Fraunhofer pattern with periods of ΔB ~ 2.8 G, which agrees with the theoretical prediction of ΔB with λ_L ~ 0.3 μm obtained in the independent measurements[2].

4. Heat dissipation by electron-phonon coupling in bilayer graphene in low temperature regime

The saturation behaviour of resistance by the dissipative Joule heating shown in Figs. S5(a), (b), and (c) gives information about the electron-phonon coupling in the bilayer graphene Josephson junction device. Crossover temperature (T_0) and the saturation resistance (R) correspond to the electron temperature in association with the base sample holder temperature and the dissipative power $P = I^2R$, respectively, with bias current I = 1 nA r.m.s. Most of the heat generated by the bias current is dissipated via electron-phonon coupling, since hot electron

diffusion into the electrodes can be ignored due to the exponentially suppressed quasiparticle density of states of the lead (Pb) superconducting electrode[3]. Also, we can assume that the phonon of bilayer graphene is fully thermalized to the temperature of the silicon oxide substrate since the interfacial thermal resistance is a few orders of magnitude smaller than the thermal resistance between electron and phonon of the bilayer graphene[3,4]. Here, the interfacial thermal resistance at low temperature is estimated by extrapolating the experimental data in Ref. [4].

Fig. S5(d) displays the relation between crossover temperature and P along with the best-fit curve of $P = A(T_{el}^{\theta} - T_{ph}^{\theta})$, giving the best-fit value of electron-phonon coupling exponent $\theta = 2.8 \pm 0.1$ for the (base) phonon temperature T_{ph} = 50 mK and the coefficient $A = 77 \pm 14$ fW·K$^{-2.8}$. Here, we assumed that the electron temperature (T_{el}) at the base temperature is saturated to T_0. The exponent $\theta = 2.7 \pm 0.1$ was also determined by the slope in double logarithmic plot in Fig. S5(e), assuming that T_{ph}^{θ} term was negligible compared to T_{el}^{θ} for T_{el} > 100 mK. The exponent θ was smaller than 4 and close to 3, which mimicked the electron-phonon coupling in disordered monolayer graphene systems in millikelvin temperature range[3,5]. This low value of the exponent ($\theta < 4$) makes bilayer graphene system a unique platform for the bias-dependent finite-size scaling studies for the independent determination of a dynamical critical exponent. This sharply contrasts with ordinary two-dimensional electron systems[6] (with $\theta = 4 - 7$), which are easily driven into 'dangerous' regime where Joule heating significantly enhances the electron temperature and thus obscures the quantum critical scaling behaviour.

5. Finite-size scaling with bias electric field

As discussed in the main text, the bias current (I) dependence of R_{sq} is also differentiated into two phases and enables finite-size analysis on electric field (E). Analysis similar to the one in Fig. 4(b) is adopted to determine the exponent $v(z+1)$, but now $(dR/dx)_{x=0}$ is plotted as a function of I as shown in Fig. S6. Using I instead of electric field $E\ (\propto IR)$ as an external parameter is valid because the resistance at SIT point is universal irrespective of I. The crossover from classical to quantum percolation with lowering I resembles the previous observation in the T-dependent scaling. More scattering of the data for smaller I is due to reduced signal-to-noise ratio in dc measurements. Here, one should be careful lest the Joule heating power $P\ (\propto E^2)$ should enhance the carrier temperature as $T_{el} \propto P^{1/\theta}$ and alter the intrinsic scaling behaviour. For the observed exponent $\theta = 2.8$ in BLG in the Section 5, our quantum critical scaling would be in the 'safety' criterion[7] of $2/\theta > z/(z+1)$ with z=1, where the self-heating effect was negligible compared with the intrinsic fluctuation effects.

6. Estimation of the number of graphene layers

To identify the number of graphene flakes, we used the intensity contrast in the green light range[8]. Fig. S6(a) shows the optical image of graphene flakes exfoliated on a highly electron-doped Si substrate capped with a 300-nm thick SiO_2 layer. Green light contrast (C_{green}) of the graphene flakes shows the linear relationship to the number of graphene layers as shown in Fig. S6(b). Bilayer graphene part (region 2) was selected to fabricate the dual-gated bilayer graphene Josephson junction device in this study.

Supplementary References

1 Tinkham, M. *Introduction to Superconductivity*. (Dover, 2004).

2 Jeong, D. *et al.* Observation of supercurrent in PbIn-graphene-PbIn Josephson junction. *Phys. Rev. B* **83**, 094503, (2011).

3 Borzenets, I. V. *et al.* Phonon bottleneck in graphene-based Josephson junctions at millikelvin temperatures. *Phys. Rev. Lett.* **111**, 027001, (2013).

4 Chen, Z., Jang, W., Bao, W., Lau, C. N. & Dames, C. Thermal contact resistance between graphene and silicon dioxide. *Appl. Phys. Lett.* **95**, 161910, (2009).

5 Chen, W. & Clerk, A. A. Electron-phonon mediated heat flow in disordered graphene. *Phys. Rev. B* **86**, 125443, (2012).

6 Chow, E., Wei, H. P., Girvin, S. M. & Shayegan, M. Phonon emission from a 2D electron gas: Evidence of transition to the hydrodynamic regime. *Phys. Rev. Lett.* **77**, 1143-1146, (1996).

7 Sondhi, S. L., Girvin, S. M., Carini, J. P. & Shahar, D. Continuous quantum phase transitions. *Rev. Mod. Phys.* **69**, 315-333, (1997).

8 Blake, P. *et al.* Making graphene visible. *Appl. Phys. Lett.* **91**, 063124, (2007).

Figures and Figure Legends

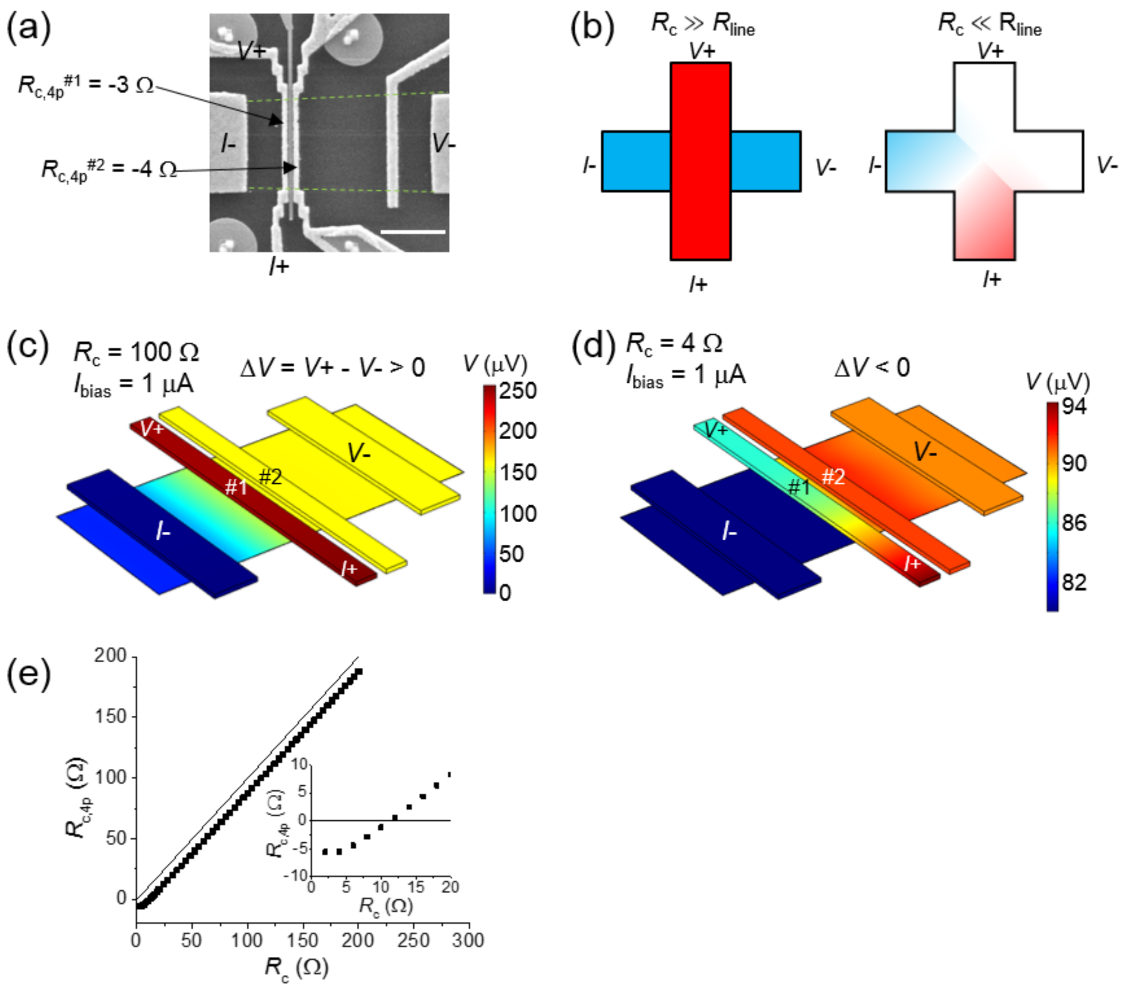

Fig. S1. Contact resistance. (a) Scanning electron microscope (SEM) image of the device with the contact-resistance measurement scheme. Green dotted lines denote the boundary of the bilayer graphene. (b) Schematics of voltage profile of high and low contact-resistance regimes. Red and blue colours represent high and low electric potential, respectively. (c, d) Numerical simulation of voltage profiles with contact resistance, R_c = 100 Ω (c) and R_c = 4 Ω (d). (e) Simulated four-probe contact resistance ($R_{c,4p}$) as a function of R_c. Solid line represents $R_{c,4p}$ = R_c. Inset, a close-up view of (e). As R_c gets lower than the electrode resistance R_{line} ~ 7 Ω, $R_{c,4p}$ can be negative.

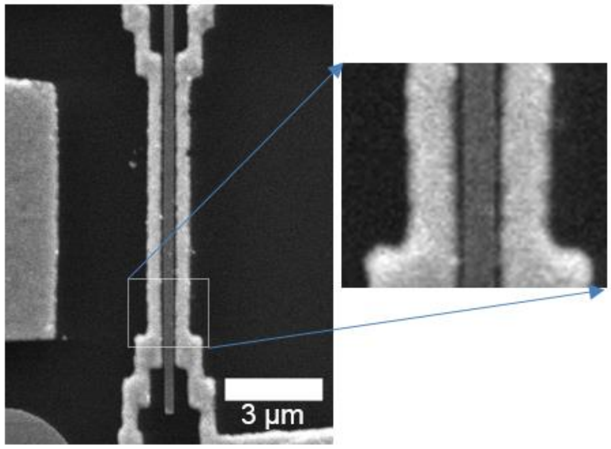

Fig. S2. Scanning electron microscope (SEM) image. SEM image of a dual-gated bilayer graphene Josephson junction device. Magnified image shows the top gate that is aligned to the superconducting electrodes as close as possible without touching them to avoid the gate leakage. The gap between the top gate and superconducting electrodes is less than 20 nm.

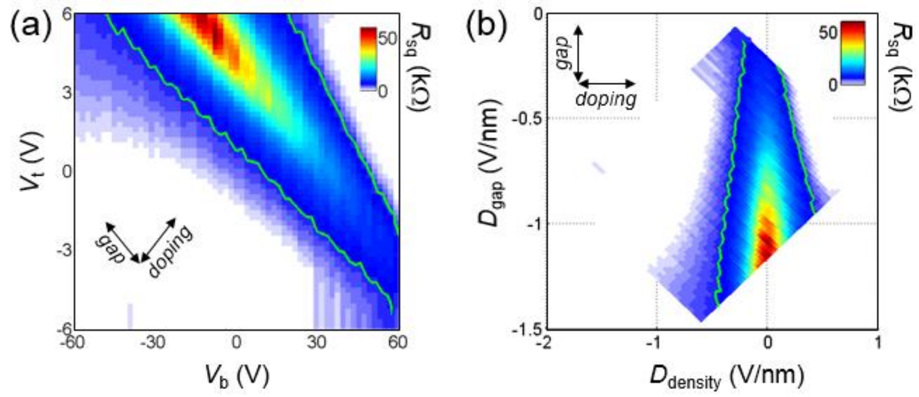

Fig. S3. Coordinate transformation. (a) Colour-coded plot of the junction resistance in (V_b, V_t) coordinate system. (b) The same in ($D_{density}$, D_{gap}) coordinate system. The green contour lines correspond to the quantum resistance of Cooper pairs, $R_Q = h/4e^2$.

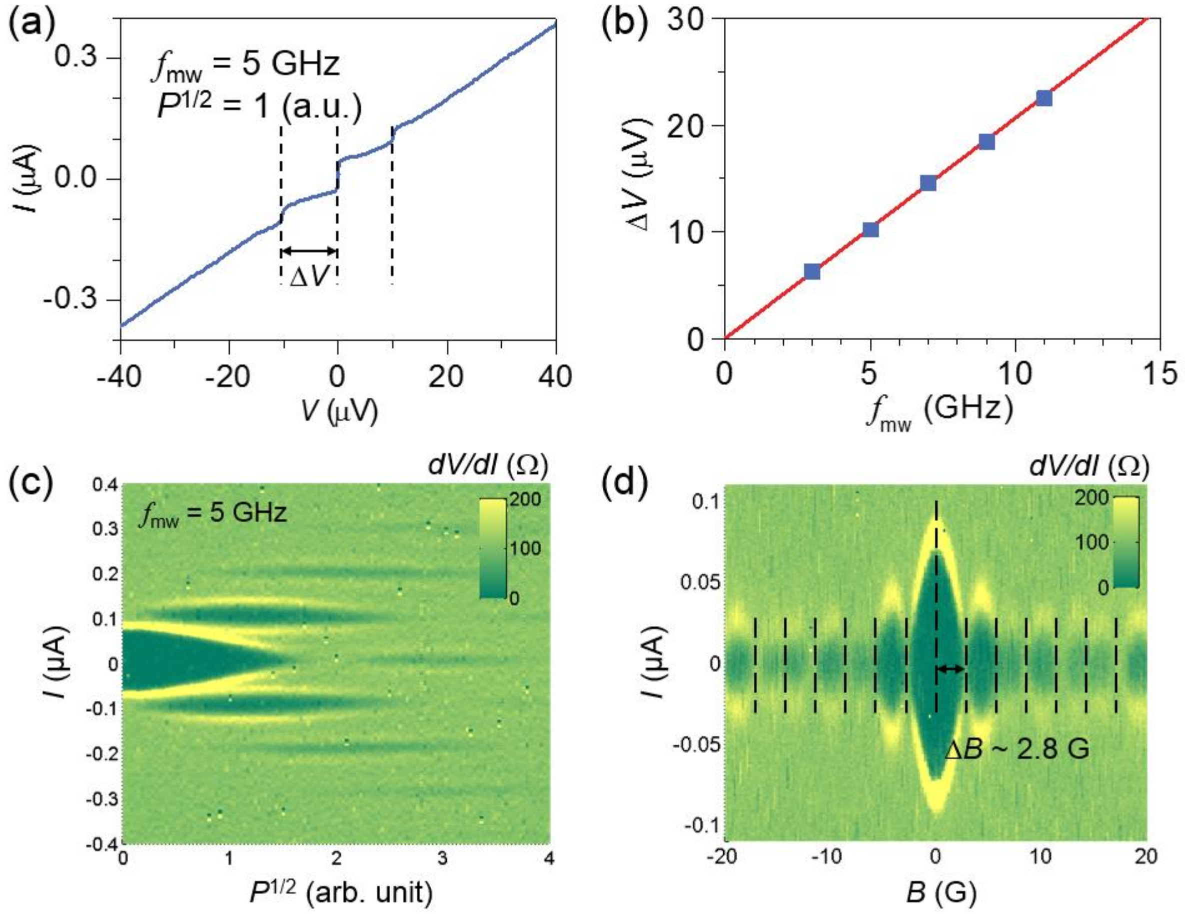

Fig. S4. Josephson coupling in the superconducting phase. (a) Equidistant voltage steps (ΔV) appear in current–voltage characteristics with microwave irradiation of frequency f_{mw} = 5 GHz. (b) f_{mw} dependence of ΔV (symbols) agrees with theoretically predicted linear relationship, $\Delta V = hf_{mw}/2e$ (red line). (c) Microwave amplitude ($P^{1/2}$) dependence of Shapiro steps at a fixed frequency f_{mw} = 5 GHz shows quasi-periodic Bessel-function-like oscillations. (d) Perpendicular magnetic field (B) dependence of the junction critical current shows the Fraunhofer pattern in constant periods of ΔB ~ 2.8 G.

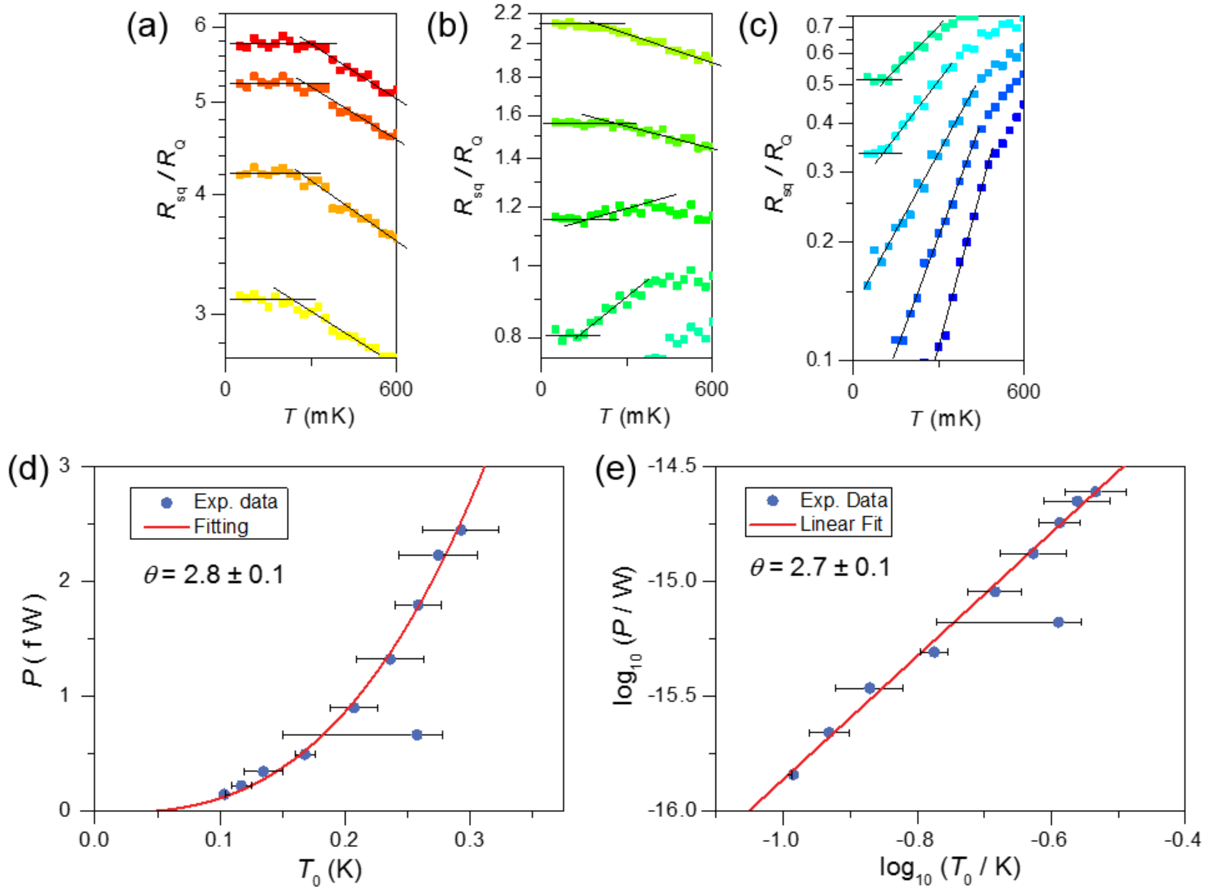

Fig. S5. Enhancement of electron temperature due to the dissipative Joule heating. Temperature dependence of square resistance (R_{sq}) divided by quantum resistance of Cooper pair (R_Q) plotted in a semi-log scale for a fixed $D_{gap} = -0.86$ Vnm^{-1} (a) at $D_{density} = 0$ (top), −0.05, −0.10, −0.15 (bottom) Vnm^{-1}, (b) $D_{density} = -0.20$ (top), −0.25, −0.30, −0.35 (bottom) Vnm^{-1}, (c) $D_{density} =$ −0.40 (top), −0.45, −0.50, −0.55, −0.60 (bottom) Vnm^{-1}. Saturation of resistance is guided by solid lines. (d) Relation between crossover temperature (T_0) and dissipative power by Joule heating (P). Solid line is the best-fit curve. (e) Log-log plot of T_0 versus P and the corresponding the best linear fit.

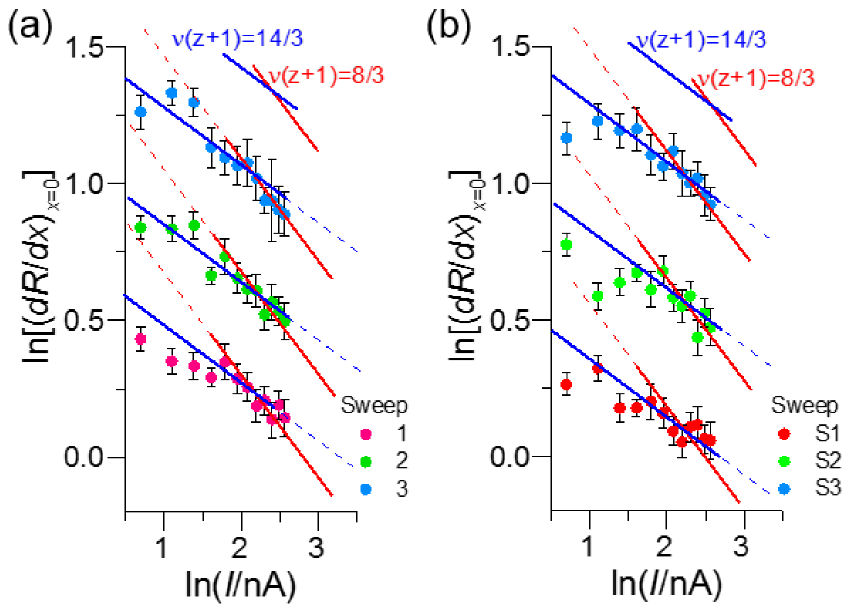

Fig. S6. Bias-current dependence of the resistance slope at the transition $(\mathrm{d}R/\mathrm{d}x)_{x=0}$ for different gate sweeps (a) 1 – 3 and (b) S1 – S3. Each set of data is plotted with an arbitrary vertical shift for clarity. Red (blue) straight line shows the expectation of the classical (quantum) percolation.

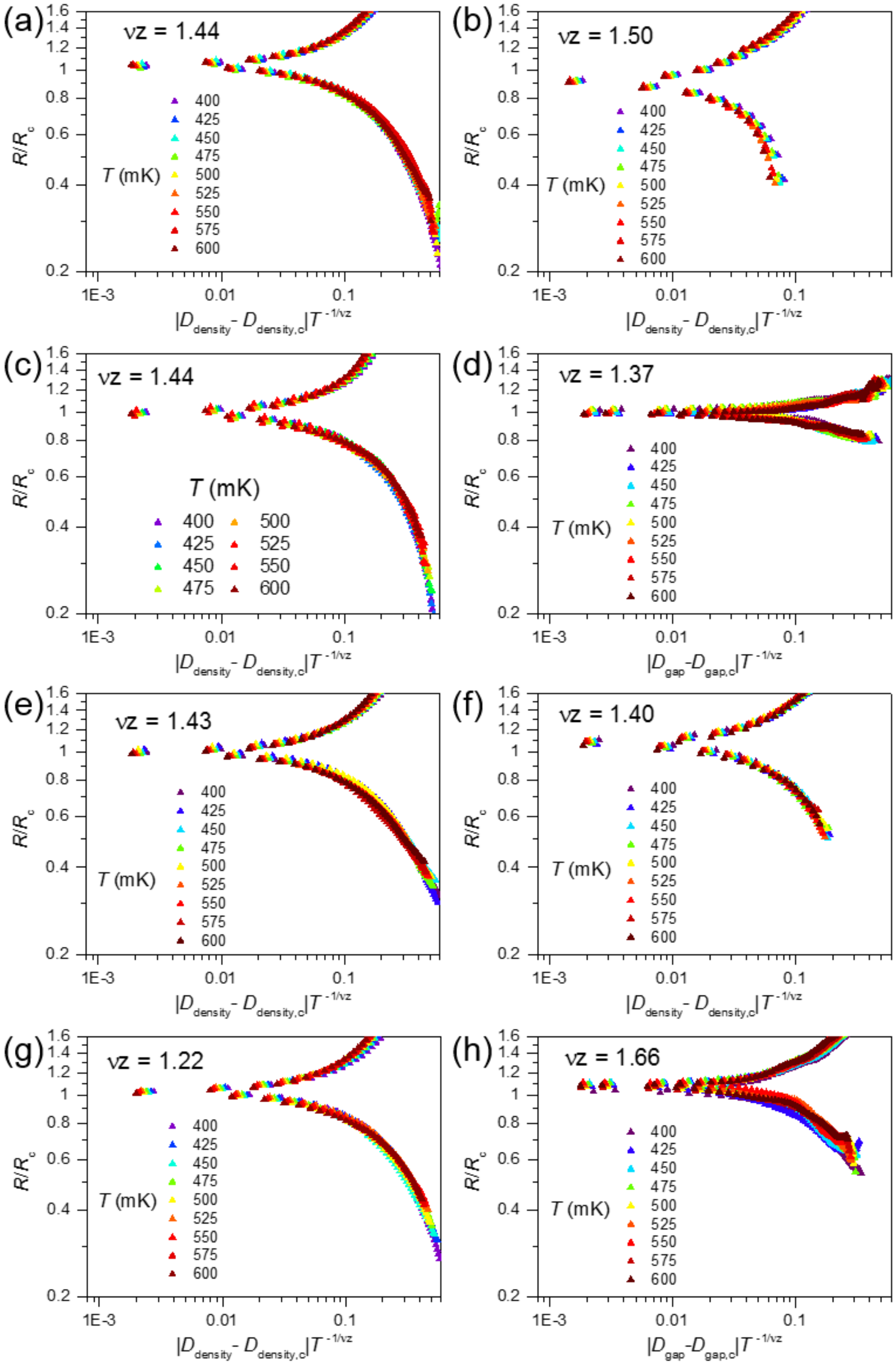

Fig. S7. Scaling analysis for temperature variation. For the sweep 1 (a) in the hole and (b) electron sides. For (c) the sweep 2 and (d) 3. For the sweep S1 (e) in the hole and (f) electron sides. For (g) the sweep S2 and (h) S3.

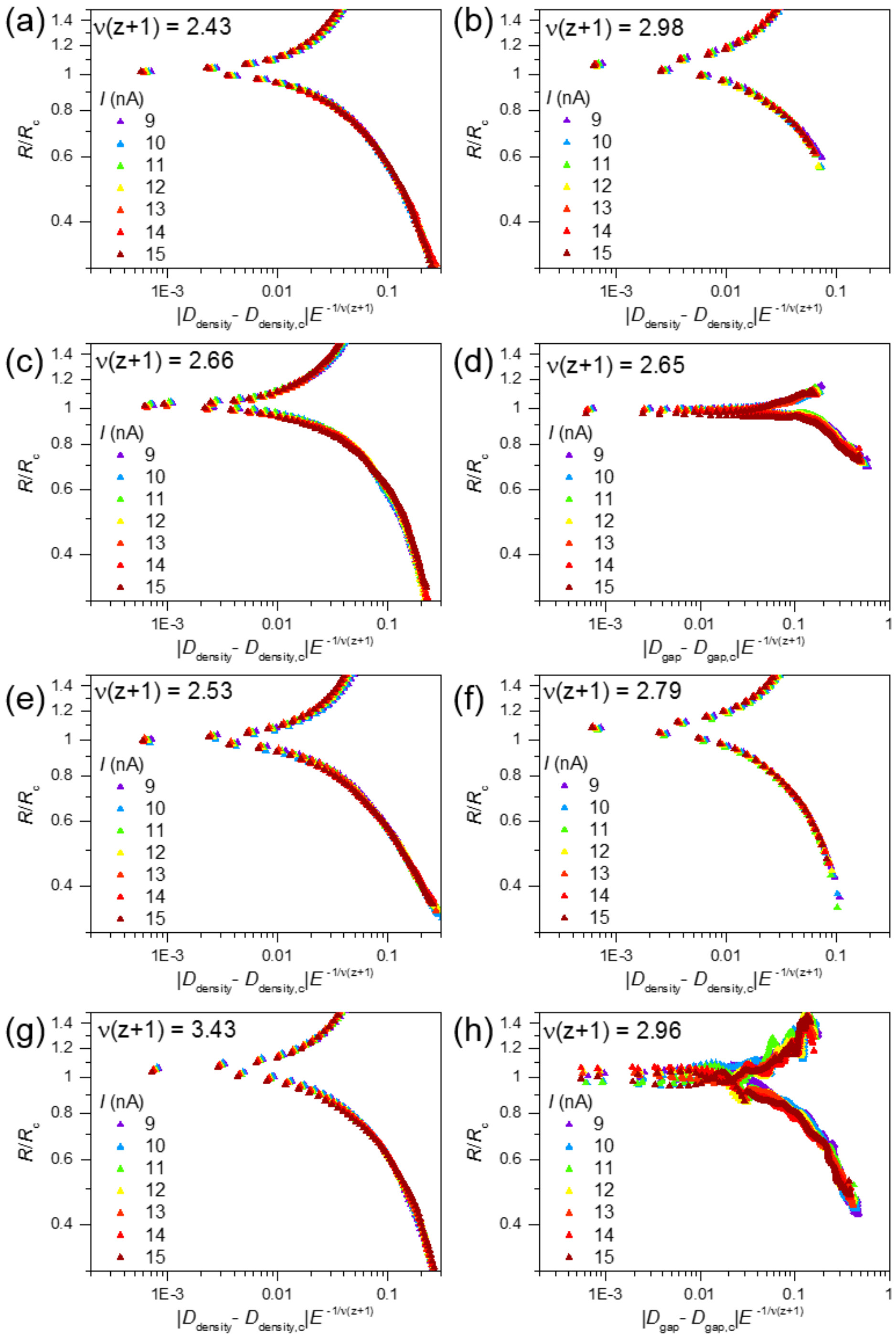

Fig. S8. Scaling analysis for electric-field variation. For the sweep 1 (a) in the hole and (b) electron sides. For (c) the sweep 2 and (d) 3. For the sweep S1 (e) in the hole and (f) electron sides. For (g) the sweep S2 and (h) S3.

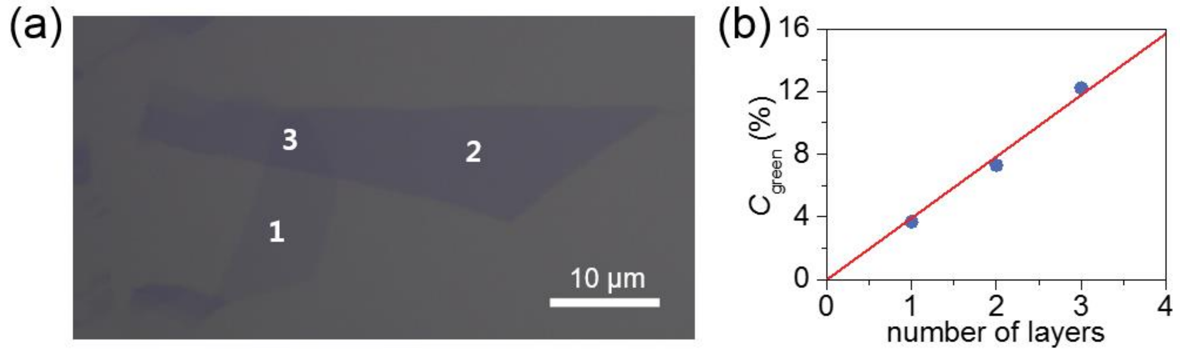

Fig. S9. Determination of the number of graphene layers. (a) Optical image of the graphene flakes exfoliated on an oxidised silicon substrate. The number of the graphene layers is denoted. (b) Linear relationship between the green-light contrast and the number of graphene layers. Red line crossing the origin is the best linear fit to the data.
